# Pituitary Adenylate Cyclase Activating Peptide (PACAP) Participates in Adipogenesis by Activating ERK Signaling Pathway

**DOI:** 10.1371/journal.pone.0072607

**Published:** 2013-09-09

**Authors:** Tatjana Arsenijevic, Françoise Gregoire, Jeanne Chiadak, Elodie Courtequisse, Nargis Bolaky, Jason Perret, Christine Delporte

**Affiliations:** Laboratory of Pathophysiological and Nutritional Biochemistry, Université Libre de Bruxelles, Brussels, Belgium; University of Rouen, France

## Abstract

Pituitary adenylate cyclase activating peptide (PACAP) belongs to the secretin/glucagon/vasoactive intestinal peptide (VIP) family. Its action can be mediated by three different receptor subtypes: PAC1, which has exclusive affinity for PACAP, and VPAC1 and VPAC2 which have equal affinity for PACAP and VIP. We showed that all three receptors are expressed in 3T3-L1 cells throughout their differentiation into adipocytes. We established the activity of these receptors by cAMP accumulation upon induction by PACAP. Together with insulin and dexamethasone, PACAP induced adipogenesis in 3T3-L1 cell line. PACAP increased cAMP production within 15 min upon stimulation and targeted the expression and phosphorylation of MAPK (ERK1/2), strengthened by the ERK1/2 phosphorylation being partially or completely abolished by different combinations of PACAP receptors antagonists. We therefore speculate that ERK1/2 activation is crucial for the activation of CCAAT/enhancer- binding protein β (C/EBPβ).

## Introduction

Obesity is an increasing disorder, in terms of incidence in the population worldwide and its extent, which is considered as a critical onset risk factor for other diseases such as cardiovascular diseases, diabetes mellitus, hyperlipidemia and cardiac infarction [1], [2]. The increase of adipose tissue mass that accompanies obesity is due to an increase in adipocyte number (hyperplasia) and size (hypertrophy) [3]. The *in vitro* 3T3-L1 preadipocytes differentiation to adipocytes represents the most useful model to study the entire adipogenic process. Upon stimulation by an appropriate combination of adipogenic signals, growth arrested 3T3-L1 preadipocytes re-enter the cell cycle progression, undergo one or two rounds of mitosis (the mitotic clonal expansion (MCE)), then exit the cell cycle and enter the terminal differentiation process [4]. After withdrawal from the cell cycle, preadipocytes start expressing adipocyte-specific genes [5], [6]. Elevation of cAMP concentration has been associated with crucial events in the early differentiation program such as induction of CCAAT/enhancer- binding protein β (C/EBPβ), that in turn triggers the expression of a number of transcription factors, like CCAAT/enhancer-binding protein α (C/EBPα), and the proliferator-activated receptor γ (PPARγ), that play essential roles in adipogenic differentiation as they promote the transcription of various genes responsible for fat transport and accumulation, such as, aquaporin 7, and adipose-specific aquaglyceroporin, upregulated by PPARγ in terminally differentiated adipocytes [5], [7]–[9].

Pituitary adenylate cyclase–activating polypeptide (PACAP) belongs to the secretin/glucagon/vasoactive intestinal peptide (VIP) family. PACAP is involved in a large array of physiological and pathophysiological processes related to development, growth, differentiation and immune responses [10]. PACAP binds and activates three different receptors belonging to the B family of G protein-coupled receptors (GPCR-B): PAC1, VPAC1 and VPAC2 [10]. The PAC1 receptor has unique affinity for PACAP, while VPAC1 and VPAC2 show equal affinity for PACAP and VIP [11]. The VPAC/PAC receptors are distributed widely throughout the body, including the respiratory system, the gastrointestinal tract, and the central nervous system [10]. Like all members of the GPCR-B family, VPAC/PAC receptors are preferentially coupled to Gαs protein that stimulates adenylate cyclase activity and induces an increase of intracellular cyclic AMP (cAMP). Coupling to phospholipase C as well as the calcium/inositol triphosphate pathway has also been described [12].

An increasing body of evidence has shown that PACAP acts on both lipid and carbohydrate metabolism [13]. For instance, PACAP enhances glucose-induced insulin secretion *in vivo* and *in vitro* [14]–[16], and insulin-stimulated glucose uptake in adipocytes [17], [18]. The ensuing hypothesis that PACAP promotes postprandial mobilization of glucose, was confirmed by impaired glucose tolerance exhibited by PAC1-deficient mice [19]. Furthermore, PACAP-knockout mice, fed normal diet, displayed reduced body mass due to a reduction in adiposity [20]. This therefore raised the hypothesis that PACAP could promote adipogenesis.

The aim of this study was to determine if PACAP can induce adipogenesis by stimulating the *in vitro* differentiation of 3T3-L1 preadipocytes into adipocytes. Herein, we showed that PACAP stimulates adipocyte differentiation, together with insulin and dexamethasone, confirmed by the elevated expression of crucial adipogenic transcription factors such as C/EBPβ, C/EBPα and PPARγ. Moreover, we showed that all three PACAP receptors, VPAC1, VPAC2 and PAC1 are present on growth-arrested undifferentiated 3T3-L1 cells. Finally, we showed that PACAP stimulation increases cAMP production within 15 min upon stimulation and induces the expression and phosphorylation of MAPK (ERK1/2), firmly supported by ERK1/2 phosphorylation being partially or completely abolished by various combinations of PACAP receptors antagonists.

## Materials and Methods

Dulbecco’s modified Eagle’s medium (DMEM, 4.5 g/l glucose), streptomycin/penicillin, fetal bovine serum, horse serum and calf serum were obtained from Invitrogen (Carlsbad, CA, USA). Bovine serum albumin, bovine insulin, 3-isobutyl-1-methylxanthine (IBMX), and dexamethasone were purchased from Sigma (St. Louis, MO, USA). PACAP27 was purchased from Bachem (Bubendorf, Switzerland). Peptidic antagonists were previously synthesized in our laboratory [21]–[23].

### Cell Culture

3T3-L1 cells, were kindly provided by Dr I. Pirson [24], and grown in DMEM supplemented with 10% calf serum, 100 U/ml penicillin and 100 mg/ml streptomycin, and in 8% CO_2_/humidified atmosphere at 37°C. Adipocyte differentiation was induced 2 days post-confluence by incubating cells for 60 h in DMEM supplemented with 10% fetal bovine serum and containing 500 µM IBMX, 0.25 µM dexamethasone and 10 µg/ml insulin (XDI cocktail) or 10^−7 ^M PACAP, 0.25 µM dexamethasone and 10 µg/ml insulin (PDI cocktail). The cells were then maintained in the same medium supplemented with insulin only. Cells were harvested at different time points: at day 0 (undifferentiated confluent cells), during mitotic clonal expansion (MCE), and during terminal differentiation (TD) up to day 9.

### Oil-Red-O Staining

Oil-Red-O staining of lipid vesicles allows substantiation of preadipocyte differentiation to adipocytes [25]. Oil-Red-O staining was carried out on day 9 after induction of differentiation. Cells were rinsed with PBS prior to fixing with 4% paraformaldehyde for 15 min. Cells were washed 3 times with PBS and then cells were incubated in an Oil- Red-O solution for 15 min (Stock solution: 0.5 g oil Red in 100 ml isopropanol, dilution 1.6× in water). Cells were then washed 3 times with water and photographed. To quantify the triglycerides content of the adipocytes, Oil-Red-O-stained adipocytes triglycerides were extracted with a mix of isopropanol and heptane (3∶2 ratio), and the absorbance was measured by spectrophotometry at 520 nm.

### cAMP Measurement

Confluent undifferentiated 3T3-L1 cells corresponding to day 0 in the differentiation protocol were grown in 3.5-cm diameter culture dishes, rinsed in Krebs-Ringer-HEPES (KRH) medium [25 mM HEPES (pH 7.4), 1.25 mM KH_2_PO_4_, 124 mM NaCl, 1.25 mM MgSO_4_, 8 mM glucose, 1.45 mM CaCl_2_, and 5 mM KCl], and preincubated with the same medium for 30 min. Following medium removal, cells were incubated in fresh KRH medium containing 1 mM IBMX and various concentrations of PACAP for 5, 15 and 30 minutes. The incubation was stopped by withdrawal of the medium and addition of 0.1 M HCl to the cells. The samples were evaporated in a Speed-Vac concentrator (Jouan RC10.10, St. Nazaire, France), and cAMP was finally measured by RIA [26].

### RNA Isolation

Cells were harvested by scrapping in 1 ml acid phenol/guanidinium thiocyanate solution (Purezol, Bio-Rad Laboratories, Hercules, CA, USA), and stored at −20°C until RNA extraction. RNA extraction was carried out using the Aurum Total RNA Fatty and Fibrous Tissue kit (Bio-Rad Laboratories, Hercules, CA, USA) according to the manufacturer’s instructions, with some minor modifications [27]. RNA concentration and purity, as well as RNA integrity, were assessed as previously described [27].

### Primer Design, cDNA Synthesis and qPCR

Primers design, cDNA synthesis and qPCR were performed as previously described [27].

### Analysis of Gene Expression Stability

Gene expression stability analysis and matching statistics were performed using Biogazelle qBASE Plus software [28]. Data were normalized using at least 3 reference genes, and the choice of reference genes used varied for MCE and TD phases as previously described [27].

### Protein Extraction and Western Blot Analysis

3T3-L1 cells from different time points of differentiation were washed with calcium- and magnesium-free PBS and lysed in 1 ml of lysis buffer containing 50 mM Tris/HCl (pH 7.5), 150 mM NaCl, 0.5% Nonidet P40, 50 mM NaF, 1 mM sodium orthovanadate, dithiothreitol and a cocktail of protease inhibitors (Complete EDTA free, Roche). Whole cell lysates were prepared and submitted to SDS-polyacrylamide gel electrophoresis (SDS-PAGE) in the presence of 5% ß-mercaptoethanol using 12% polyacrylamide gels. Proteins were transferred to polyvinyldiene difluoride membranes and immunolabeled using primary antibodies against VPAC1, VPAC2, PAC1, cdk4, cdk2, cyclin D3, cyclin A (Santa Cruz Biotechnology Inc., Santa Cruz, CA), ERK1/ERK2, phospho ERK1/ERK2, PPARγ (Cell Signalling, Danvers, MA, U.S.A.), β-actin and aquaporin 7 (Millipore). The bound primary antibodies were detected using secondary anti-mouse or anti-rabbit antibodies (GE Healthcare, Little Chalfont, Buckinghamshire, U.K.) and ECL chemiluminescence detection kit (Perkin Elmer, Waltham, MA, U.S.A.).

### Statistical Analysis

Data are presented as mean ± SEM. Group means were compared by repeated measure ANOVA and Tukey’s, Dunnett’s or Boniferroni’s comparison tests. Differences were considered statistically significant at p<0.05.

## Results

### PACAP can Induce Adipogenesis together with Insulin and Dexamethasone

**Figure 1:**
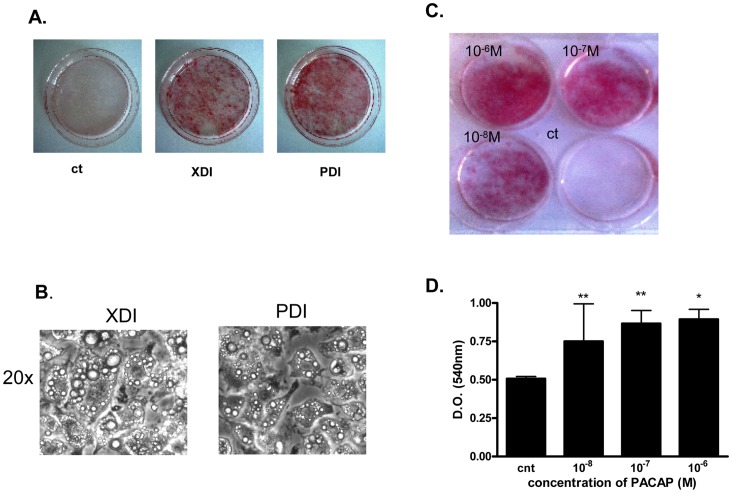
Bright field and Oil-Red-O staining photographs of 3T3-L1 cells. A. 2 days post-confluent cells were induced to differentiate by treatment with 500 µM IBMX, 0.25 µM dexamethasone and 10 µg/ml insulin (XDI cocktail) or 10^−7 ^M PACAP, 0.25 µM dexamethasone and 10 µg/ml insulin (PDI cocktail), as described under Material and Methods. The cells were stained with Oil-Red-O and photographed on day 9. B. Micrographs of 3T3-L1 cells induced to differentiate with IBMX, dexamethasone and insulin (XDI) and PACAP, dexamethasone and insulin (PDI). C. Oil-Red-O staining of cells induced to differentiate with increasing concentrations of PACAP (10^−8^ M to 10^−6^ M), 0.25 mM dexamethasone and 10 µg/ml insulin. Control cells were maintained in medium without induction with hormones. D. Triglyceride content in cells induced to differentiate with increasing concentrations of PACAP (10^−8 ^M to 10^−6^ M), 0.25 mM dexamethasone and 10 µg/ml insulin. Data were analyzed using repeated measure of ANOVA and by Dunnett’s comparison tests. *p<0.05 compared to control.

To investigate the effects of PACAP on preadipocyte differentiation into adipocytes, 2 days post-confluent 3T3-L1 cells were kept for 60 h either in control medium (ct), or in medium containing IBMX, dexamethasone and insulin (XDI) or 10^−7 ^M PACAP, dexamethasone and insulin (PDI). Thereafter, cells were then kept in medium with insulin only and the differentiation process was followed up to 9 days. Cells stimulated with XDI or PDI differentiated into adipocytes, as evidenced by Oil-Red-O staining at day 9, indicating the accumulation of intracellular triglycerides (Figure 1A) and by the appearance of microscopic multilobular fat droplets (Figure 1B). In contrast, control cells, kept only in medium without hormonal stimulation, did not enter the differentiation process, remaining unstained by Oil-Red-O (Figure 1A). The effect of PACAP on adipocyte differentiation of 3T3-L1 cells was dose-dependent as shown again by Oil-Red-O staining and triglyceride content quantification after Oil-Red-O extraction (Figures 1C and 1D)**.**

### PACAP Containing Differentiation Cocktail (PDI) Induced the Expression of Adipocyte Differentiation Markers

**Figure 2:**
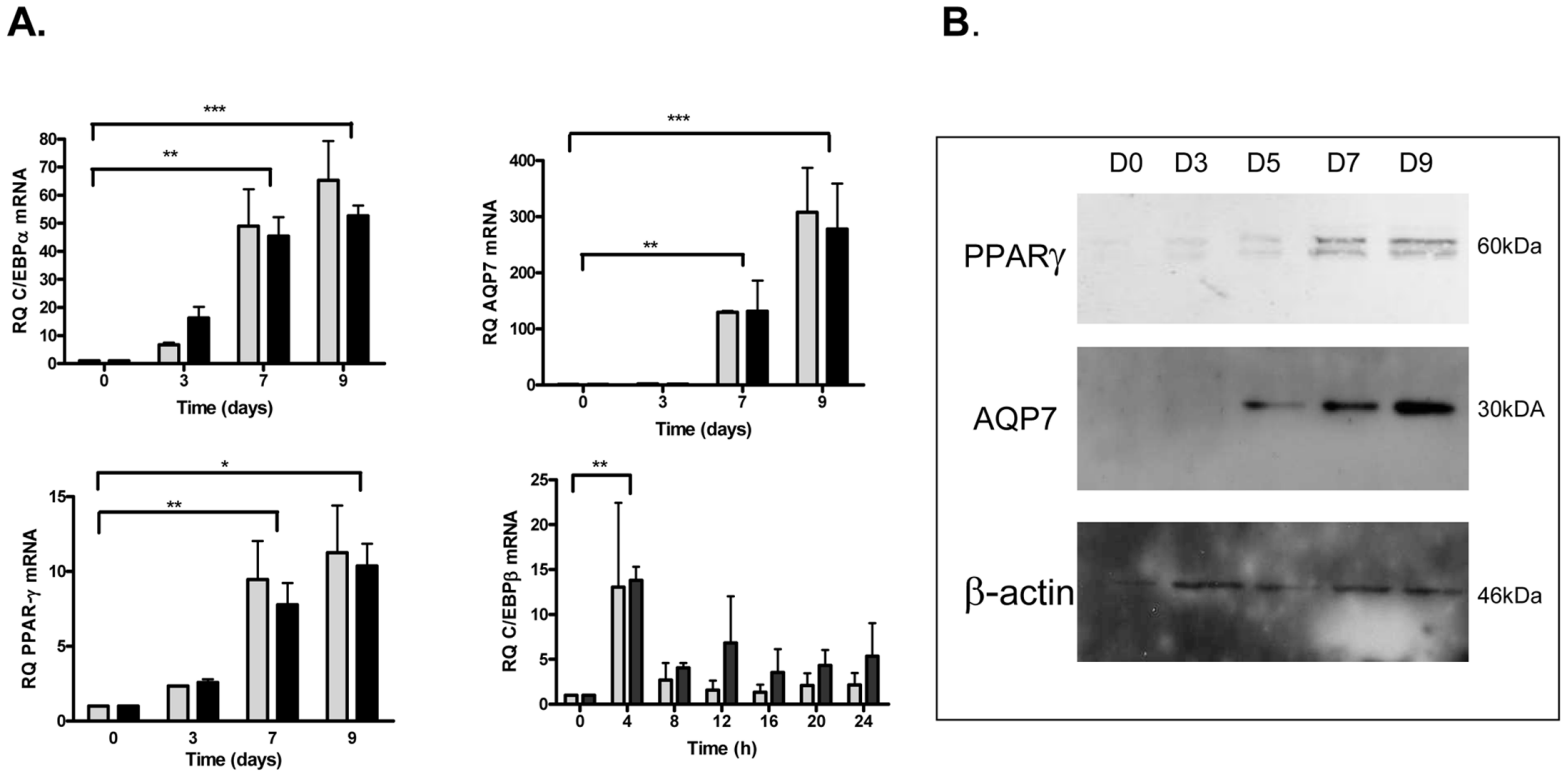
Expression of C/EBPα/β, PPARγ and aquaporin 7 (AQP7) during differentiation of 3T3-L1 cells by XDI and/or PDI cocktail. A. Quantification of mRNA expression levels of crucial transcription factors, C/EBPα/β, PPARγ and AQP7 a PPARγ target, at the indicated times of differentiation in mouse 3T3-L1 adipocytes. Cells were induced to differentiate with XDI (shaded) or PDI (black) hormonal cocktail. C/EBPα, PPARγ and AQP7 mRNA expression levels were measured by qPCR during TD till day 9. cEBPβ mRNA expression levels were measured during MCE. Data were analyzed using repeated measure of ANOVA and by Tukey’s comparison tests. *p<0.05; **p<0.01; ***p<0.001 (XDI and PDI compared to day 0). B. Protein expression levels of PPARγ and AQP7 during the indicated time points of differentiation with PDI cocktail.

The expression of transcription factor C/EBPβ, C/EBPα, PPARγ mRNAs and of AQP7 mRNA, a target of PPARγ, was assessed by qPCR in 3T3-L1 cells submitted to differentiation by either PDI or XDI stimulation (Figure 2A). PACAP at a concentration of 10^−7 ^M, together with dexamethasone and insulin, significantly increased the expression of C/EBPα (±60 fold) and PPARγ (±12 fold) mRNAs at both days 7 and 9, and noteworthy AQP7 mRNA (±120 fold at day 7, and ±300 fold at day 9), with amplitude and kinetics similar to the classical differentiation cocktail XDI. Furthermore, the expression of C/EBPβ mRNA, an early marker of adipocyte differentiation in response to cAMP, was strongly upregulated at 4 h in response to both XDI and PDI. The upregulation of PPARγ and AQP7 during differentiation was confirmed by Western blot analysis (Figure 2B). Due to the lack of appropriate antibodies against C/EBP proteins, protein expression could not be monitored.

### Synchronous Cell Cycle Reentry upon Induction of Differentiation

**Figure 3:**
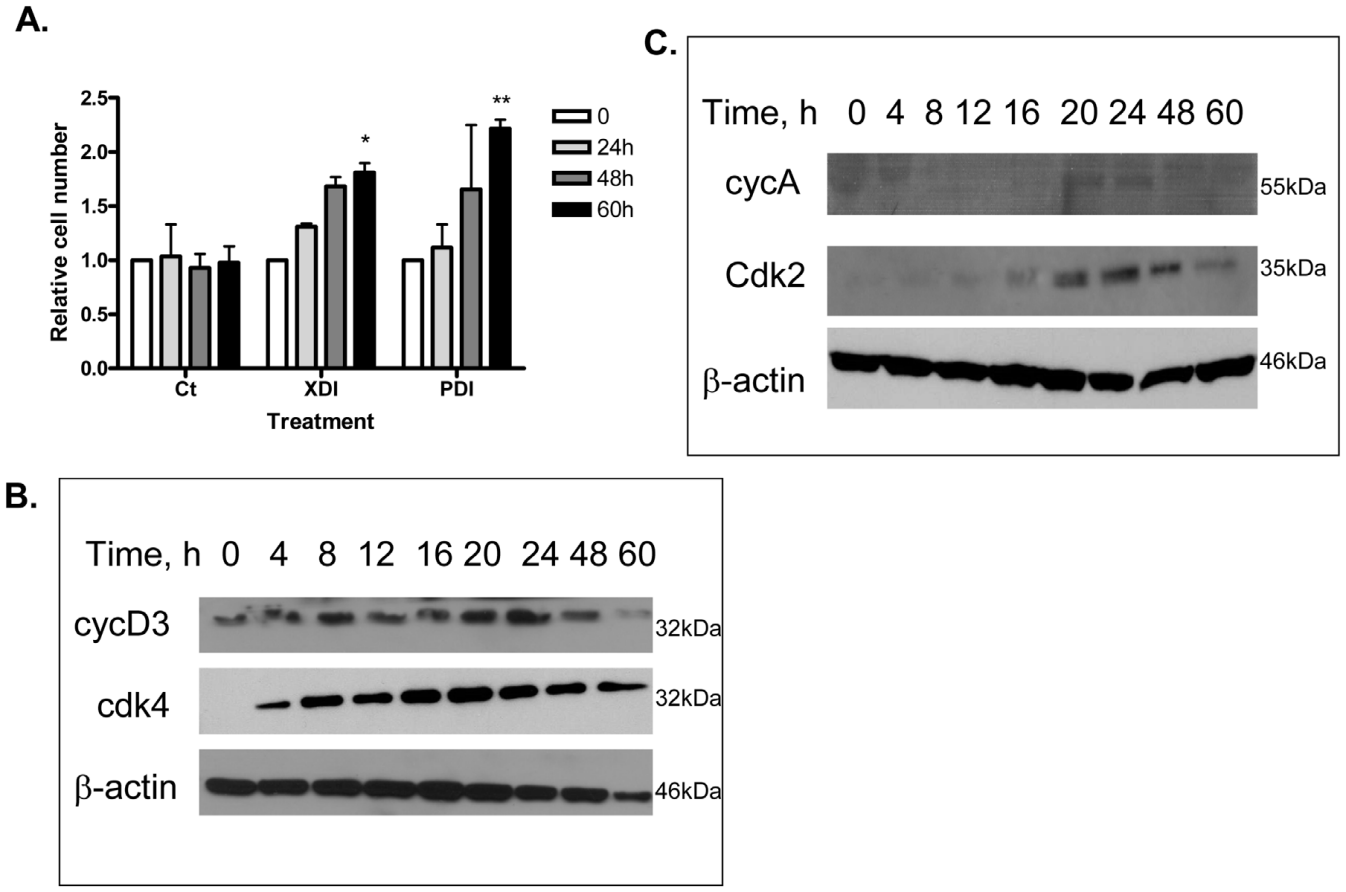
MCE analysis of 3T3-L1 cells induced to differentiate by PDI cocktail. A. Synchronous reentry of the cell cycle by growth arrested 2 days post-confluent 3T3-L1 preadipocytes after induction of differentiation with XDI or PDI hormonal cocktail. Control cells (Ct) were kept in medium without hormonal stimulation. Cell number was determined using a Coulter counter and relative cell number was plotted. Data were analyzed using repeated measure of ANOVA and by Dunnett’s comparison tests. *p<0.05; **p<0.01 B and C. Changes in the expression of cell-cycle proteins during MCE. Two days post-confluent cells were induced to differentiate into adipocytes by the PDI cocktail. At the times indicated, whole cell lyzates (40 µg) were subjected to SDS/PAGE and immunoblotted.

Two days post-confluent, growth arrested 3T3-L1 cells maintained in the presence of control medium did not proliferate over 60 h. However, when growth arrested 3T3-L1 cells were induced to differentiate with XDI, they underwent one sequential round of mitosis that was completed by 60 h (Figure 3A). Similarly to XDI, in our experimental set-up, PDI induced, statistically significant, 2 fold increase in cell number (one round of mitosis) over 60 h. The proliferation of PDI-stimulated cells was analyzed further by assessing the expression of proteins involved in the cell cycle by Western blot. As evidenced by the expression of cyclin D3 and cdk4 (Fig. 3B), growth-arrested cells were in G1 phase at the time of induction of differentiation. Consistent with the entry into S phase, expression of cdk2 and cyclin A increased between 12 and 16 h and were then maintained at that level up to 48 h (Figure 3C).

### Expression of PAC1, VPAC1, and VPAC2 during 3T3-L1 Adipogenesis

**Figure 4:**
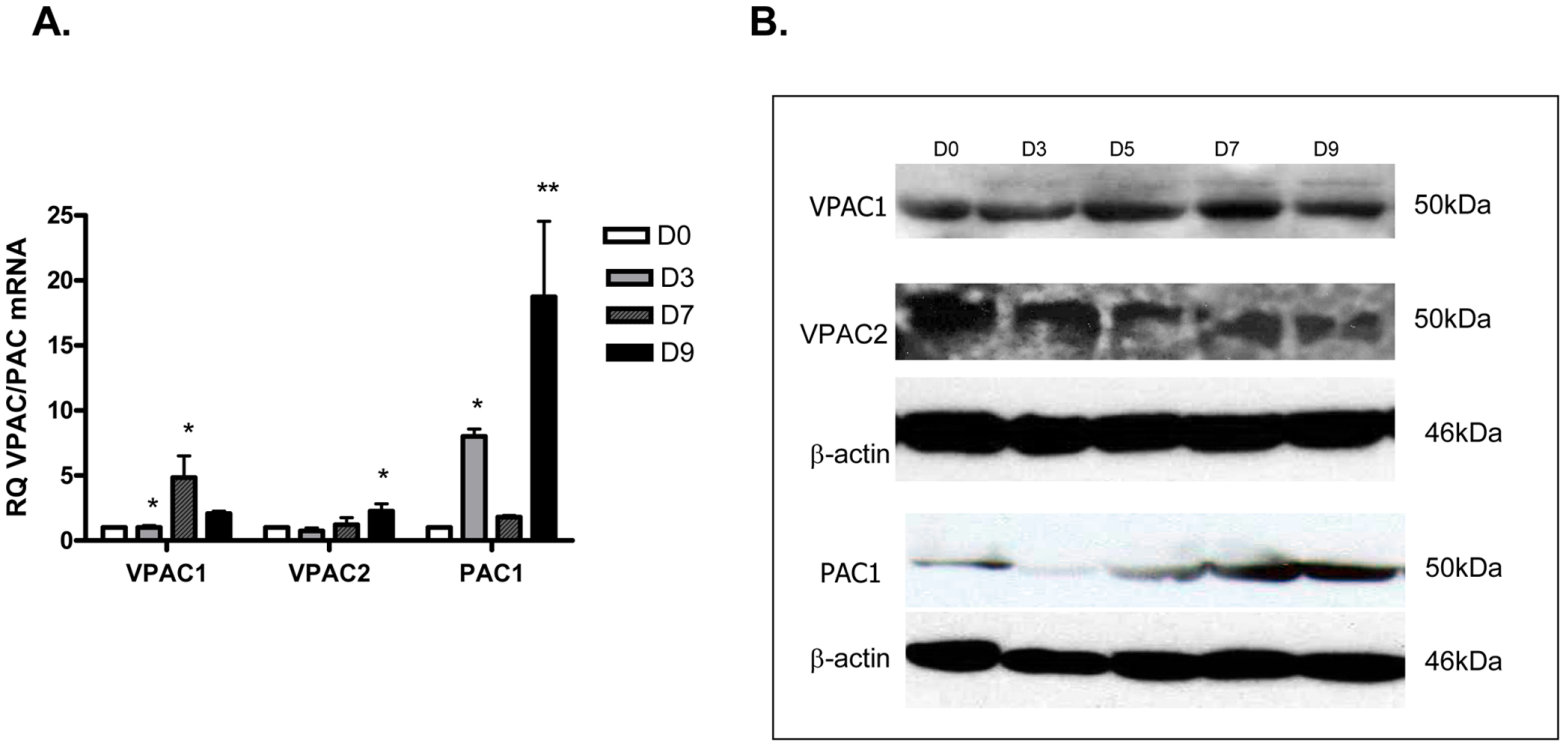
Expression of VPAC/PAC receptors in 3T3-L1 cells during differentiation of 3T3-L1 cells. **A.** qPCR analysis of the mRNA expression of VPAC/PAC receptors in 3T3-L1 two days post-confluent cells (day 0) and during differentiation till day 9. Data were analyzed using repeated measure of ANOVA and by Dunnett’s comparison tests. *p<0.05; **p<0.01 compared to day 0. **B.** Protein expression levels of VPAC/PAC receptors during differentiation of 3T3-L1 cells.

In order to determine which of the three VPAC/PAC receptors contributed to the adipogenic effect of PACAP, the expression of the receptors was investigated during the entire differentiation process of 3T3-L1 cells into adipocytes. Undifferentiated cells corresponding to day 0 and cells corresponding to successive differentiation stages (from day 3 up to day 9) were subjected to qPCR (Figure 4A) and Western blotting analysis (Figure 4B). qPCR analysis showed expression of VPAC1, VPAC2 and PAC1 mRNAs in both undifferentiated 3T3-L1 cells and 3T3-L1 cells differentiated into adipocytes. VPAC1 mRNA expression was significantly upregulated during differentiation at days 5 (p<0.05) and 7 (p<0.05), compared to day 0. The expression of VPAC2 mRNA was significantly increased during differentiation at day 9, compared to day 0 (p<0.05). PAC1 mRNA was significantly upregulated during the adipogenesis process at days 5 (p<0.05) and 9 (p<0.01), compared to day 0 (Fig. 4A). Western blotting analysis confirmed the presence of the VPAC/PAC receptors in undifferentiated cells. PAC1 protein level was upregulated during the adipogenesis process, while the expression levels of both VPAC1 and VPAC2 proteins remained steady during the entire process (Fig. 4B).

### PACAP Induced Accumulation of cAMP in 3T3-L1 Fibroblasts

**Figure 5:**
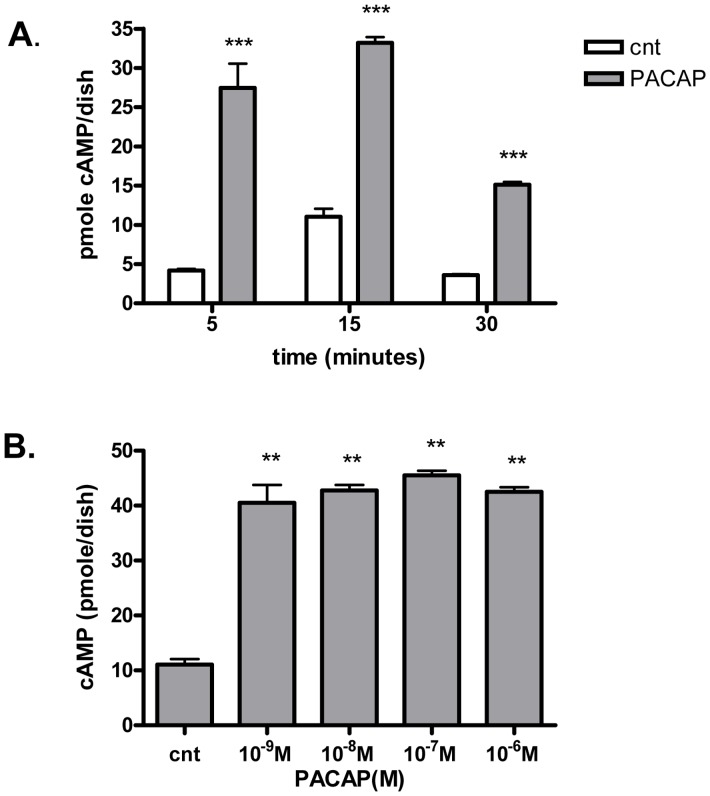
cAMP accumulation in 3T3-L1 preadipocytes exposed to PACAP measured by RIA. A. 2 days post-confluent cells were rinsed and preincubated for 30 min with KRH medium at 37°C. The medium was removed and cells were incubated for 5, 15 or 30 min with fresh medium containing IBMX (white) and 10^−7 ^M PACAP (shaded). Data were analyzed using repeated measure of ANOVA and by Dunnett’s comparison tests. ***p<0.001, compared to IBMX. B. Cells were incubated for 15 min with fresh medium containing IBMX and various concentrations of PACAP. Data were analyzed using repeated measure of ANOVA and by Boniferroni comparison tests. **p<0.01, compared to control.

cAMP content was measured by RIA in 2 days post-confluent 3T3-L1 cells submitted or not to 10^−7 ^M PACAP for various times (Figure 5). Following PACAP stimulation, cAMP accumulation occurred as early as 5 min, reached a maximum at 15 min, and decreased as of 30 min (Figure 5A). Following 15 min incubation, increasing concentrations of PACAP induced a biphasic dose-response accumulation curve of cAMP in 3T3-L1 cells, with a maximum for 10^−7 ^M PACAP (Figure 5B).

### PACAP Induced ERK1/2 Phosphorylation

**Figure 6:**
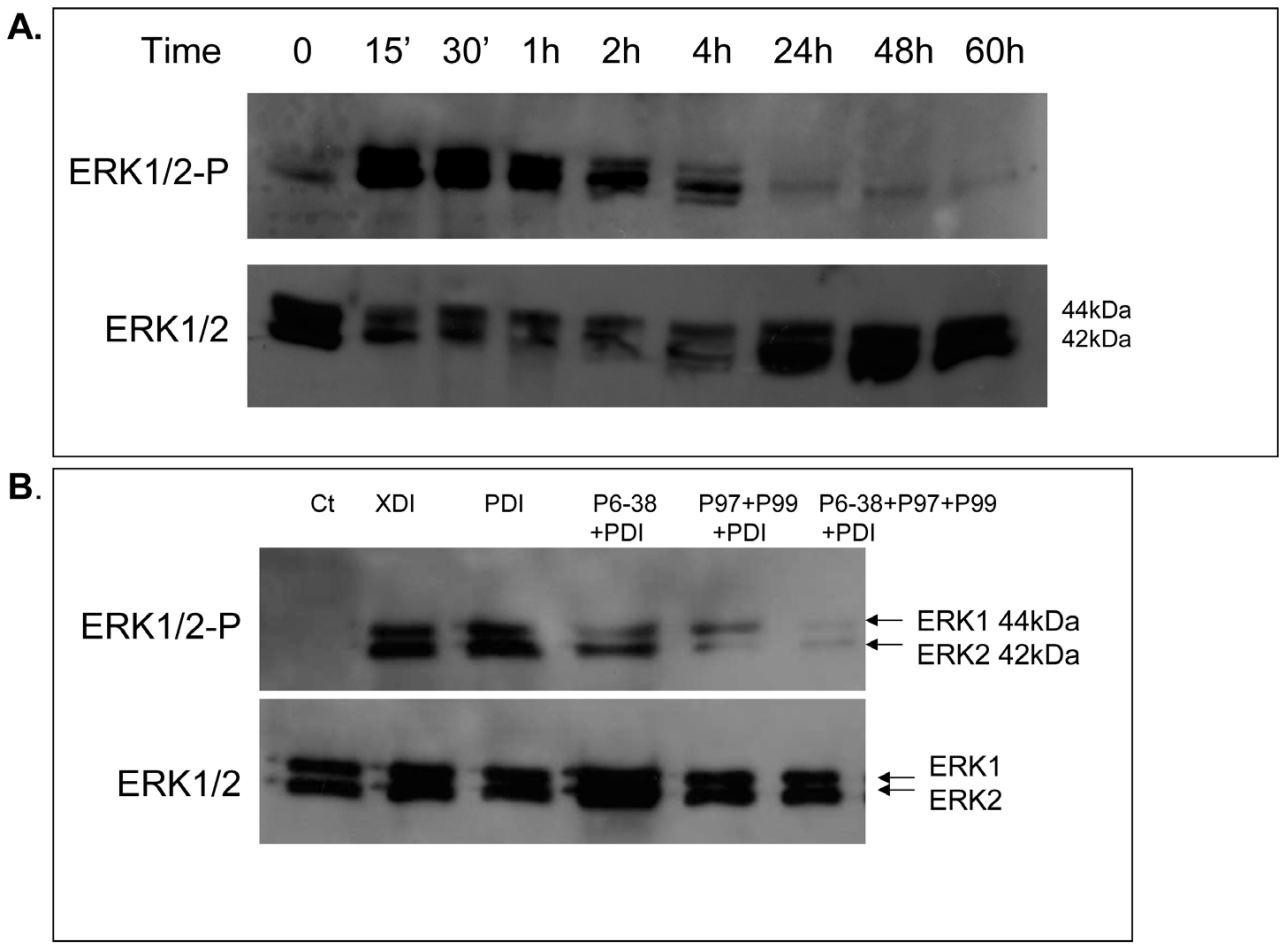
Effects of PDI cocktail on ERK1/2 expression and activation during MCE. A. 2 days post-confluent cells were induced to differentiate with the PDI cocktail and whole cell lysates were prepared at indicated times during MCE. 40 µg of whole cell lysate were subjected to SDS/PAGE analysis and immunoblotted with phospho- ERK1/2 (ERK1/2-P) and total ERK1/2 antibody. B. 2 days post-confluent cells were exposed to different combination of VPAC/PAC antagonists (10^−7 ^M) prior to stimulation with the PDI cocktail for 30 minutes. 40 µg of whole cell lysates were subjected to SDS/PAGE analysis and immunoblotted with phospho- ERK1/2 (ERK1/2-P) and total ERK1/2 antibody.

It has been shown that MEK/ERK signaling pathway regulates the expression of the adipogenic transcription factors during the early phase of adipogenesis [29]. In order to evaluate if PACAP could be involved in ERK1/2 activation during MCE, ERK1/2 phosphorylation and total protein expression were analyzed in response to PDI stimulation of cells (Figure 6A). PDI stimulation of cells induced rapid and massive activation of ERK1/2, as indicated by its ability to phosphorylate ERK1 and ERK2 (Figure 6A). To test whether PACAP is directly implicated in the activation of ERK1/2, cells were pretreated with VPAC/PAC antagonists prior to stimulation with PDI. Both cells treated with XDI and PDI induced the activation of ERK1/2 (Figure 6B). In cells pretreated with PAC1 specific antagonist (PACAP 6-38), prior to PDI stimulation, the phosphorylation of ERK1 was strongly suppressed. When cells were pretreated with a combination of VPAC1/2 antagonists (PG-97269) specific for VPAC1 and PG-99465, specific for VPAC2), phosphorylation of both ERK1 and ERK2 was suppressed, though the effect on ERK2 was more pronounced. When cells were preincubated with the combination of all 3 antagonists (PACAP 6-38, PG-97269, PG-99465) prior to PDI stimulation, ERK1/2 phosphorylation was nearly completely abolished.

## Discussion

Adipocytes undergo lipogenesis and lipolysis for the storage and release of energy to meet the needs of the body. Adipocyte-dependent physiological functions become pathophysiological when adipocytes develop excessively, as it becomes a risk factor that may lead to diseases such as metabolic syndrome and diabetes [30]. Signals affecting adipocyte differentiation and function are currently of considerable interest. PACAP has been shown to be lipogenic by potentiating insulin-dependent glucose uptake [17], and lipolytic in the absence of insulin [31]. The present study demonstrates for the first time a positive implication of PACAP, together with insulin and dexamethasone on the adipogenesis of 3T3-L1 cells. Our results suggest that PACAP exerts its adipogenic role via cAMP accumulation and subsequent ERK1/2 activation early upon induction of adipogenesis.

The role of ERK1/2 in adipogenesis remained controversial for a long time before it became clear that ERK pathways can both, promote and inhibit adipogenesis, depending on kinetics of activation during the differentiation process [29]. MEK activation, which is rapidly induced during the initial hours following induction of differentiation of 3T3-L1 cells, facilitates the expression of adipogenic transcription factors, while MEK1 activation at subsequent times might inhibit adipogenesis. Our results show that PDI strongly activates ERK1/2 activity during the initial hours upon induction of differentiation (4 h), but subsequently decreases it during MCE (from 24 h on). Recent studies suggest that ERK1 is involved in adipogenesis as ERK1−/− knockout animals have decreased adiposity and fewer adipocytes than wild-type animals. Furthermore ERK1−/− cells had impaired adipogenesis that was unaffected by inhibitors of ERK pathway, suggesting that ERK2 is not implicated in adipocyte differentiation [32].

It has been shown that all three PACAP receptor types are present in human adipose tissue [33]. In the present study, 3T3-L1 pre-adipocytes and adipocytes expressed PAC1 receptors in addition to VPAC1 and VPAC2 receptors. During adipogenesis, the expression of VPAC1 mRNA reached a maximum at day 7, while the expression of VPAC2 was higher at day 9. The significance of the particular biphasic time course of PAC1 mRNA expression, increasing at days 5 and 9 but not at day 7, will require further investigations. Our work strongly indicates that the PAC1 receptor signaling could lead to ERK1 phosphorylation. However, an assumption has been made on the actual specificity of the antagonist. Previous work by our group and by others, questions the total specificity of the antagonist used. Indeed, the VPAC2 antagonist PG-99465 has previously been shown to have weak agonistic effects on VPAC1 [21]. The PAC1 antagonist PACAP 6–38 has previously been shown to act antagonistically on VPAC2 in addition to PAC1 [34].

Under the current state of knowledge pertaining to the antagonist specificity, it is not possible to assess which of the three receptors, or combination thereof, are implicated in the induction of specific genes via increased levels of cAMP, known to bind to the cAMP response element in specific promoters. The cAMP-dependent increase of C/EBPβ in the early stages of adipocyte conversion induces an elevated expression of C/EBPα, and PPARγ, [35], [36]. Our qPCR results confirmed the upregulation of PPARγ, C/EBPα and β expression in 3T3-L1 adipocytes induced to differentiate by PDI. These results are in agreement with the qPCR analysis of adipose tissue from PACAP−/− KO mice, where a significant reduction in PPARγ expression is observed [37]. We also showed a strong upregulation of C/EBPβ mRNA expression in cells stimulated with PDI. This latter observation is notably supported by in astrocyte cultures, wherein PACAP induces C/EBP transcription factors expression, including C/EBPβ and C/EBPδ [38].

Although expression of C/EBPβ occurs rapidly upon induction of differentiation (within 2 h), acquisition of DNA binding activity and transcription of PPARγ and C/EBPα are delayed. Previous studies have shown that C/EBPβ is sequentially phosphorylated during 3T3-L1 differentiation [39]. Phosphorylation on Thr188 that occurs in G1 phase by MAPK was shown to prime C/EBPβ for subsequent phosphorylation and cdk2/cyclinA maintains this phosphorylated state throughout S phase and MCE, until the subsequent phosphorylation by GSK3β that induces DNA-binding and transactivation activity of C/EBPβ [40]. As we show a direct effect of PACAP on the activation of ERK1/2, early during MCE, we speculate that PACAP may induce differentiation by activation of C/EBPβ via ERK1/2-induced phosphorylation of C/EBPβ on Thr188. Further work is however still necessary to address this hypothesis.

In conclusion, we showed for the first time that PACAP can positively regulate adipogenesis together with insulin and dexamethasone in 3T3-L1 adipocytes. The present study demonstrated that PACAP via induction of cAMP acts on early steps in adipogenesis by activating ERK1/2, event that contributes to the activation of a transcription factors cascade that regulates adipogenesis. PACAP, known as an insulinotropic, plays a role in adipogenesis, suggesting that PACAP can potentiate insulin action in adipogenesis.
